# Surgical treatment improves overall survival of hepatocellular carcinoma with extrahepatic metastases after conversion therapy: a multicenter retrospective study

**DOI:** 10.1038/s41598-024-60379-x

**Published:** 2024-04-28

**Authors:** Xiaoshi Zhang, Xiaodong Zhu, Jianhong Zhong, Yang Zhao, Xiaoyun Zhang, Wenwen Zhang, Feng Ye, Chaoxu Yang, Jun Xue, Rui Xiong, Jiabei Wang, Shunli Shen, Yangxun Pan, Dongxiao Li, Tianqiang Song, Xinyu Bi, Huichuan Sun, Bangde Xiang, Shanzhi Gu, Tianfu Wen, Shichun Lu, Yongjun Chen, Tao Yin, Lianxin Liu, Ming Kuang, Li Xu, Deyu Li, Jianqiang Cai

**Affiliations:** 1 Department of Hepatobiliary Surgery, National Cancer Center/National Clinical Research Center for Cancer/Cancer Hospital, Chinese Academy of Medical Sciences and Peking Union Medical College, Beijing, China; 2 Department of Liver Surgery and Transplantation, Zhongshan Hospital Affiliated of Fudan University, Shanghai, China; 3 Hepatobiliary Surgery Department, Guangxi Medical University Cancer Hospital, Guangxi, China; 4 Department of Interventional Therapy, Hunan Cancer Hospital Affiliated of Xiangya School of Medicine, Hunan, China; 5 Department of Liver Surgery, West China Hospital of Sichuan University, Sichuan, China; 6 Faculty of Hepato-Pancreato-Biliary Surgery, Chinese PLA General Hospital, Beijing, China; 7 Department of General Surgery, Ruijin Hospital Affiliated of Shanghai Jiao Tong University School of Medicine, Shanghai, China; 8 Department of Medical Oncology, Nanjing Jinling Hospital, Jiangsu, China; 9 Cancer Center, Union Hospital Tongji Medical College of Huazhong University of Science and Technology, Shanghai, China; 10 Department of Hepatobiliary and Pancreatic Surgery, Hubei Cancer Hospital, Tongji Medical College, Huazhong University of Science and Technology, Hubei, China; 11 Department of Hepatobiliary Surgery, The First Affiliated Hospital of USTC, Anhui, China; 12 Department of Hepatic Surgery, The First Affiliated Hospital of Sun Yat-Sen University, Guangdong, China; 13 Department of Liver Surgery, Sun Yat-Sen University Cancer Center, Sun Yat-Sen University, Guangzhou, China; 14 Department of Gastroenterology, Henan Provincial People’s Hospital, People’s Hospital of Zhengzhou University, Henan, China; 15 Key Laboratory of Cancer Prevention and Therapy, Tianjin Medical University Cancer Institute and Hospital, National Clinical Research Center for Cancer, Tianjin’s Clinical Research Center for Cancer, Tianjin, China; 16 Department of Hepatobiliary Surgery, National Cancer Center/National Clinical Research Center for Cancer/Cancer Hospital, Chinese Academy of Medical Sciences and Peking Union Medical College, Panjiayuan, Chaoyang District, in the South, 17th, Beijing, 100021 China

**Keywords:** Hepatocellular carcinoma, Extrahepatic metastases, Surgical treatment, Overall survival, Diseases, Medical research, Oncology

## Abstract

Systemic therapy is typically the primary treatment choice for hepatocellular carcinoma (HCC) patients with extrahepatic metastases. Some patients may achieve partial response (PR) or complete response (CR) with systemic treatment, leading to the possibility of their primary tumor becoming resectable. This study aimed to investigate whether these patients could achieve longer survival through surgical resection of their primary tumor. We retrospectively collected data from 150 HCC patients with extrahepatic metastases treated at 15 different centers from January 1st, 2015, to November 30th, 2022. We evaluated their overall survival (OS) and progress-free survival (PFS) and analyzed risk factors impacting both OS and PFS were analyzed. Patients who received surgical treatment had longer OS compared to those who did not (median OS 16.5 months vs. 11.3 months). However, there was no significant difference in progression-free survival between the two groups. Portal vein invasion (P = 0.025) was identified as a risk factor for poor prognosis in patients, while effective first-line treatment (P = 0.039) and surgical treatment (P = 0.005) were protective factors. No factors showed statistical significance in the analysis of PFS. Effective first-line treatment (P = 0.027) and surgical treatment (P = 0.006) were both independent protective factors for prolonging patient prognosis, while portal vein invasion was an independent risk factor (P = 0.044). HCC patients with extrahepatic metastases who achieve PR/CR with conversion therapy may experience longer OS through surgical treatment. This study is the first to analyze the clinical outcomes of patients receiving surgical treatment for HCC with extrahepatic metastases.

## Introduction

Hepatocellular carcinoma (HCC) is the 5th most common cancer and the 3rd leading cause of cancer-related deaths worldwide, with over half a million deaths annually^1,2^. Due to the high incidence of hepatitis and liver cancer in China, HCC causes the most tumor-related deaths after lung cancer^3,4^. Unfortunately, HCC is often diagnosed at an advanced stage, as it has an insidious onset and lacks obvious symptoms in the early stages, making radical surgery impossible for most patients^5,6^. Despite significant progress in targeted therapy, immunotherapy, interventional therapy and radiotherapy in recent years, the prognosis of patients with advanced liver cancer remains unsatisfactory. HCC with extrahepatic metastases should be classified as stage C according to the Barcelona Clinic Liver Cancer (BCLC) staging and systemic treatment is necessary for those patients. In China, the Chinese Liver Cancer Stage (CNLC) staging is widely used due to its applicability compared to the BCLC stage system^7^. For HCC patients with extrahepatic metastases (i.e., CNLC stage IIIb), systemic therapy is still the preferred treatment, according to the Guidelines for the Diagnosis and Treatment of Hepatocellular Carcinoma (2019 Edition)^8^. Some patients with advanced HCC have benefited from alternative conversion treatment, including the combination of targeted therapy, immunotherapy, interventional therapy and radiotherapy, leading to a significantly improved prognosis. For some patients who achieved partial response (PR) or complete response (CR) in intrahepatic lesion with systemic treatment, their primary tumor achieved a significant stage reduction and surgical resection became possible. However, there is currently a lack of relevant studies worldwide to determine whether resection of the primary tumor results in longer survival in this group of patients.

Thus, we conducted a real-world study to investigate the effect of surgical resection after effective systemic treatment in HCC patients with extrahepatic metastases. Since surgery is not a standard treatment option for advanced HCC, the number of patients who received surgical treatment was limited. Therefore, we conducted a multiple-center study in conjunction with several hospitals in China to collect as many eligible patients as possible. In this study, we compared the overall survival (OS) and progress-free survival (PFS) between patients who underwent surgery after conversion therapy and those who did not receive surgery. We also analyzed the prognostic factors associated with those patients who achieved PR/CR after conversion therapy, seeking to provide convincing medical evidence for the treatment of HCC patients who had extrahepatic metastases.

## Methods

### Patients

Ethical approval for this study (No. B2022-195R) was provided by Institutional Ethics Committee on 21 April, 2022. This work has been reported in line with the STROCSS criteria^9^. All patients were diagnosed with HCC according to the AASLD criteria without prior anti-tumor treatment. The eligibility criteria were BCLC-C stage with extrahepatic metastasis (equally to CNLC-IIIb). According to the criteria, patients were supposed to receive systemic conversion therapy with or without locoregional therapies, including transcatheter arterial chemoembolization (TACE), hepatic artery infusion chemotherapy (HAIC) and radiotherapy. Tumor response of intrahepatic lesions was evaluated as PR/CR per modified RECIST. Patients who achieved PR/CR were considered for surgical resection based on tumor resectability, patient’s general condition, and absence of contraindications for surgery.The baseline clinical data, tumor response, follow-up data were completed. Exclusion criteria included patients with other primary malignancies or a history of malignant tumor, patients with a Child C grade of liver function or whose primary tumor was evaluated as unresectable, and patients with missing follow-up data.

### Treatment and outcome

**Figure 1:**
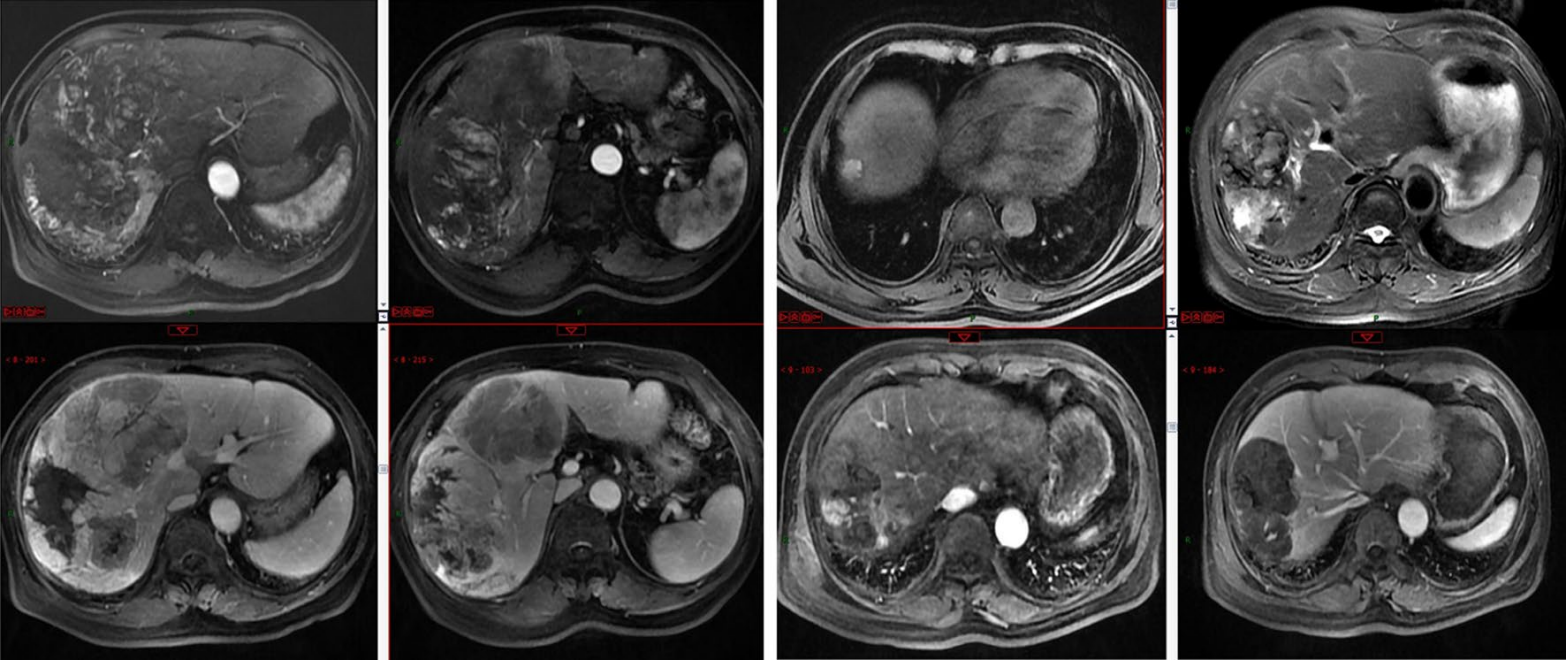
MRI image of a patient with stage IIIb HCC who achieved PR after conversion therapy. First Row (Pre-treatment MRI Scans): First: Pre-treatment axial MRI scan showing a large intrahepatic lesion with arterial phase hyperenhancement. Second: Venous phase MRI scan before treatment, highlighting the extent of the lesion with venous phase hyperintensity and the relatively decreased portal supply. Third: Delayed phase pre-treatment MRI scan, indicating a lesion with delayed phase fast-out intensity. Fourth: T2-weighted MRI scan before treatment, with the portal vein tumor thrombosis exhibiting high signal intensity. Second Row (Post-treatment MRI Scans): First: Arterial phase MRI scan after initial conversion therapy, demonstrating decreased lesion enhancement. Second: Venous phase MRI post-conversion therapy, showing partial response with reduced venous phase hyperintensity and the recovery of portal supply. Third: Delayed phase MRI after conversion therapy, revealing a decrease in lesion size with delayed phase hyperintensity. Fourth: T2-weighted MRI scan post-conversion therapy, showing a reduction in lesion size and elimination of portal vein tumor thrombosis.

Tumor response was evaluated based on the modified RECIST. Each patient was evaluated by experienced and specialized imaging specialists who were unaware of this study subgroup, at centers participated in this study. Figure 1 shows the imaging of a patient with who achieved PR after pre-operative treatment. When PR/CR in the intrahepatic tumor was achieved, surgical resection was suggested after thorough discussion with patients, though this was not a treatment option explicitly recommended by the guidelines yet. The surgery was performed after an informed consent was obtained from the patient or patients’ family, by experienced hepatobiliary surgery specialists at each center. All patients in operation group achieved R0 resection of their primary tumors. However, the patients who did not received resection but continued with the treatment regimen until tumor progression or presence of intolerable adverse effect. It is worth mentioning that the time point at which patients first achieved PR/CR after conversion therapy was chosen as the starting point for this study, which makes it easier and more accurate to compare the survival status of the two groups of patients. The response to systemic treatment was evaluated using the modified Response Evaluation Criteria in Solid Tumors (RECIST). On average, patients underwent systemic treatment for 6 months, with individual durations ranging from 3 to 12 months based on their response and tolerance to the therapy. For patients achieving partial response (PR) or complete response (CR), the management of extrahepatic lesions was customized according to the patient’s disease presentation and response to systemic therapy. Treatment options included, but were not limited to, radiofrequency ablation, stereotactic body radiotherapy, and surgical resection when appropriate. The decision to consider surgical resection in patients who achieved PR/CR after systemic treatment was based on several factors, including tumor resectability, the patient’s general condition, and the absence of contraindications for surgery. These criteria were determined through thorough discussions with the patients and their families, as well as comprehensive assessments by experienced hepatobiliary surgery specialists at each participating center. The decision to address extrahepatic lesions was made within a multidisciplinary team, considering factors such as the patient’s overall health, the extent of metastatic spread, and the response of these lesions to systemic therapy. Conversion therapy involves a multimodal treatment strategy aimed at downstaging advanced HCC to a resectable state. This approach combines systemic therapies like targeted therapy and immunotherapy with locoregional treatments to achieve a substantial reduction in tumor burden.

### Follow-up strategy

All patients included in this study had complete follow-up information with the cut-off time for follow-up set at Nov 30th, 2022. The median follow-up period was 22 months, with a range of 6 to 84 months. After achieving PR/CR with pre-operative treatment, the patients received CT or MRI exams every 2–3 months until 1 year after surgery.

### Statistical analysis

SPSS (ver. 22.0) and R (ver. 4.1.3) was used for statistical analysis. Categorical data were analyzed by chi-square test or Fisher exact test. Measures conforming to normal distribution were expressed as mean ± standard deviation while non-normally distributed measures were expressed as median (interquartile spacing). Normal data were analyzed using the t-test, and non-normal data were analyzed using the Wilcox on rank sum test. The Kaplan–Meier method was used for survival analysis. The COX model was used for univariate and multifactorial analyses. Differences were considered statistically significant at P < 0.05. All results were double-counted three times.

### Statement of ethics

The study was approved by the Institutional Ethics Committee of Zhongshan Hospital Affiliated of Fudan University (reference number: B2022-195R). Written informed consent was obtained for all patients participated in this study. All procedures were done in accordance with the Declaration of Helsinki. Patient privacy was accurately protected in this study.

## Results

### Baseline characteristics of the cohort

**Figure 2:**
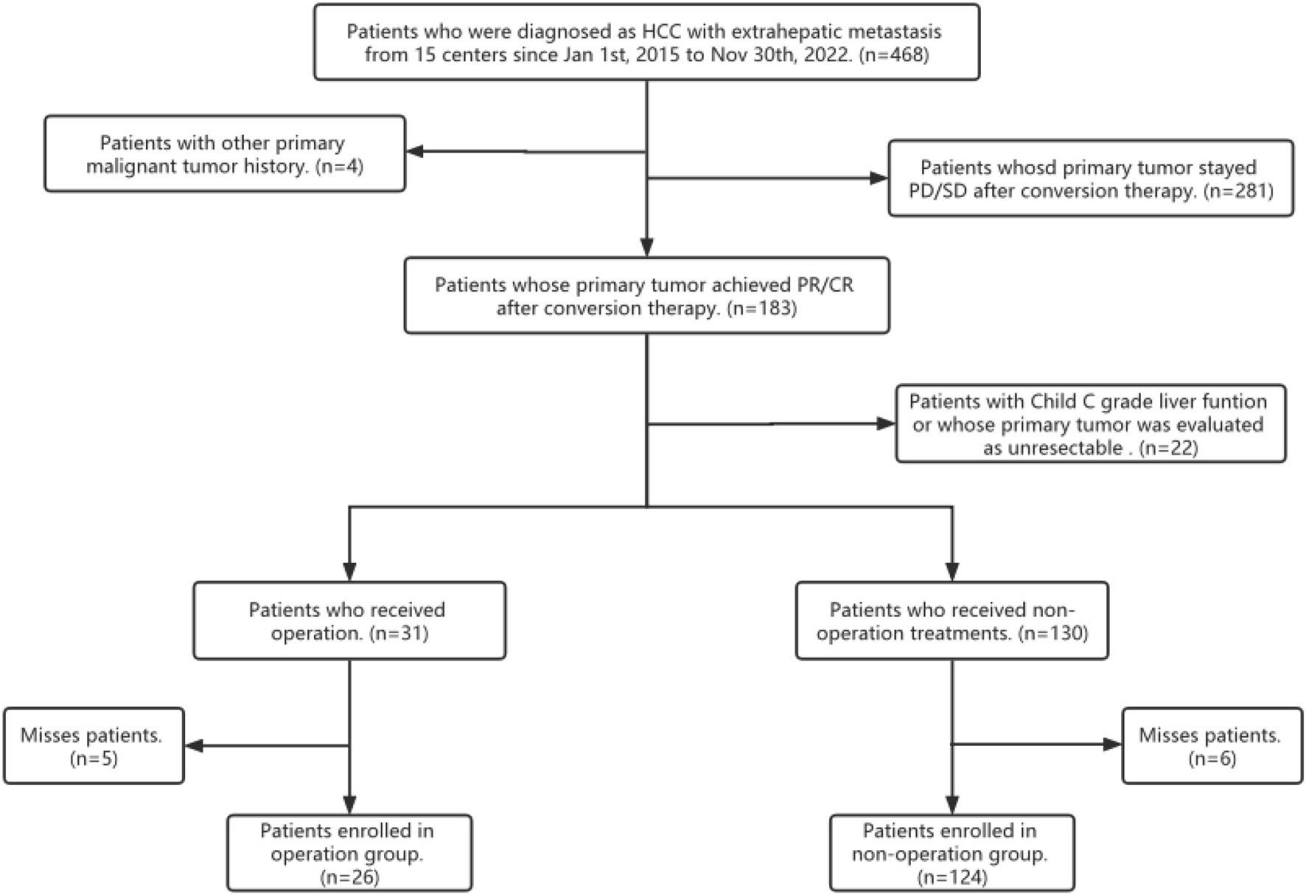
Process of screening for included patients.

A total of 150 HCC patients with extrahepatic metastases who were treated from 15 centers in China from Jan 1st, 2015 to Nov 30th, 2022 were eligible for this study. The process for inclusion of patients is shown as Fig. 2. The first-line systemic therapies include targeted therapy (sorafenib, lenvatinib, bevacizumab, etc.) and immunotherapy (sintilimab, atezolizumab, etc.). 50 patients received TACE or HAIC, radiotherapy or combination therapy to achieve a better tumor response. Although most of the patients achieved PR/CR with first-line therapy, 31 patients remain insensitive and required second-line therapy (regorafenib, nivolumab, etc.) to achieve satisfactory outcomes.

They were divided into two groups according to whether they received surgical treatment or not. The surgical group consisted of 26 patients, while the non-surgical group consisted of 124 patients, out of the 26 patients in the Operation group, 18 underwent resection of the primary liver tumor only, while 8 patients had both the primary liver tumor and the metastatic lesions surgically removed. Operation group had a higher proportion of patients with oligometastases, defined as having 1–3 metastatic lesions, which were amenable to surgical resection. Specifically, 12 out of 26 patients in the Operation group were classified as having oligometastases, compared to 28 out of 124 patients in the Non-operation group. This difference was statistically significant and suggests that patients with fewer metastatic lesions were more likely to be considered for surgical treatment. The median number of metastatic lesions in the Operation group was 2, while in the Non-operation group it was 4.Age, gender, hepatitis infection, metastatic site, BCLC stage, combined portal vein thrombosis, concomitant hepatic vein invasion, first-line treatment, size and number of primary tumor at the beginning of the study were recorded. The baseline characteristics of the two groups is shown in Table 1. And there was no statistically significant difference between the two groups, indicating that both groups were in similar conditions regarding their general health and tumor status at the start of this study, after achieving PR/CR with conversion therapy. Among the patients in the Operation group, 15% achieved Complete Response (CR) and 85% achieved Partial Response (PR) in their metastatic lesions. In contrast, the Non-operation group had 10% with CR, 75% with PR, and 15% with Stable Disease (SD). There were no patients with Progressive Disease (PD) in either group at the time of surgical consideration.What must be made clear is that there are two patients with BCLC stage D, although the general condition was poor combined with the high surgical risk, due to emergency events such as tumor rupture in the course of treatment, the primary tumor was removed at the strong request of the patients and their families. Fortunately, their operations were successful. In order to ensure authenticity, these two patients were also included in this study. Except that, all patients’ primary tumors were evaluated as resectable in both groups by complete specialist diagnostic opinions.

**Table 1:** Characteristics of the patients enrolled (n = 150).

		Operation(n = 26)	Non-operation(n = 124)	P value
Gender	Male	22 (84.6%)	110 (88.7%)	0.519
	Female	4 (15.4%)	14 (11.3%)	
Age(years)		55.0 (46.3, 61.5)	55.0 (49.8, 61.0)	0.49
HBV infection	Yes	24 (92.3%)	107 (86.3%)	0.53
	No	2 (7.7%)	17 (13.7%)	
AFP				0.346
	< 400 ng/ml	5(19.2)	36(29.0)	
	≥ 400 ng/ml	21(80.8)	88(71.0)	
Site of metastases	Lymph node	13 (50.0%)	57 (46.0%)	0.829
	Lung	5 (19.2%)	40 (32.3%)	0.242
	Bone	1 (3.8%)	22 (17.7%)	0.129
	Adrenal gland	3 (11.5%)	8 (6.5%)	0.405
	Abdominal cavity	7 (26.9%)	23 (18.5%)	0.418
BCLC stage	stage C	24 (92.3%)	120 (96.8%)	0.278
	stage D	2 (7.7%)	4 (3.2%)	
Portal vein invasion	Yes	10 (38.5%)	58 (46.8%)	0.519
	No	16 (61.5%)	66 (53.2%)	
Hepatic vein invasion	Yes	6 (23.1%)	15 (12.1%)	0.385
	No	20 (76.9%)	109 (87.9%)	
First-line treatment	Yes	20 (76.9%)	99 (79.8%)	0.791
	No	6 (23.1%)	25 (20.2%)	
Use TACE/HAIC	Yes	10 (38.5%)	40 (32.3%)	0.648
	No	16 (61.5%)	84 (67.7%)	
Diameter of primary tumor(cm)		5.5 (4.6, 7.7)	6.2 (4.8, 8.1)	0.681
Number of primary tumor	Single	17 (65.4%)	92 (74.2%)	0.468
	Multiple	9 (34.6%)	32 (25.8%)	

### Risk factors analysis

In order to identify relevant factors affecting the prognosis of HCC patients with extrahepatic metastases, we analyzed data on the general condition, tumor status and treatment options of the patients. Factors that affected OS and PFS are shown in Tables 2 and 3, respectively. The results shows that portal vein invasion is a risk factor for poor prognosis of patients, while effective first-line treatment and surgical treatment are protective factors for OS. However, no factors showed statistical significance for PFS. The multifactorial analysis for OS shows that both effective first-line treatment and surgical treatment were independent protective factors, while portal vein invasion was an independent risk factor (Table 4).

**Table 2:** Univariate analyses of overall survival in CNLC stage IIIb HCC patients (n = 150).

	OR (95%CI)	P value
Gender (Male)	2.123 (0.663–6.799)	0.205
Age over 60 years	1.017 (0.574–1.803)	0.953
With HBV infection	1.125 (0.509–2.486)	0.77
Lymph node metastasis	0.934 (0.552–1.581)	0.799
Lung metastasis	1.493 (0.856–2.603)	0.158
Bones metastasis	0.902 (0.425–1.912)	0.788
Adrenal gland metastasis	0.782 (0.244–2.508)	0.679
Abdominal cavity metastasis	0.917 (0.481–1.746)	0.791
BCLC stage C	0.747 (0.318–1.756)	0.504
With portal vein invasion	1.831 (1.080–3.104)	0.025
With hepatic vein invasion	0.570 (0.206–1.580)	0.28
Effective first-line treatment	0.541 (0.302–0.968)	0.039
Receive surgical treatment	0.188 (0.059–0.602)	0.005

**Table 3:** Univariate analyses of progress-free survival in CNLC stage IIIb HCC patients (n = 150).

	OR (95%CI)	P value
Gender (Male)	0.600 (0.266–1.352)	0.217
Age over 60 years	1.314 (0.687–2.515)	0.409
With HBV infection	1.252 (0.491–3.193)	0.639
Lymph node metastasis	1.358 (0.744–2.479)	0.318
Lung metastasis	0.427 (0.180–1.016)	0.054
Bones metastasis	0.366 (0.113–1.186)	0.094
Adrenal gland metastasis	0.942 (0.290–3.058)	0.921
Abdominal cavity metastasis	0.939 (0.444–1.985)	0.869
BCLC stage C	0.696 (0.268–1.804)	0.456
With portal vein invasion	1.255 (0.688–2.287)	0.459
With hepatic vein invasion	0.842 (0.329–2.156)	0.72
Effective first-line treatment	0.651 (0.327–1.295)	0.221
Receive surgical treatment	1.011 (0.503–2.030)	0.976

**Table 4:** Multivariate analyses of overall survival in CNLC stage IIIb HCC patients (n = 150).

	OR (95%CI)	P value
With portal vein invasion	1.720 (1.104–2.919)	0.044
Effective first-line treatment	0.517 (0.288–0.927)	0.027
Receive surgical treatment	0.195 (0.061–0.626)	0.006

### Survival analysis

The survival analysis was conducted using the Kaplan–Meier model to assess patient outcomes in both treatment groups. In the surgical group, patients exhibited a median overall survival (OS) of 16.5 months and a median progression-free survival (PFS) of 14.2 months. Conversely, patients in the non-surgical group had a median OS of 11.3 months and a median PFS of 9.5 months. Notably, progression occurred in 40.7% (11 out of 27) of patients in the surgical group compared to 25.8% (32 out of 124) in the non-surgical group.

**Figure 3:**
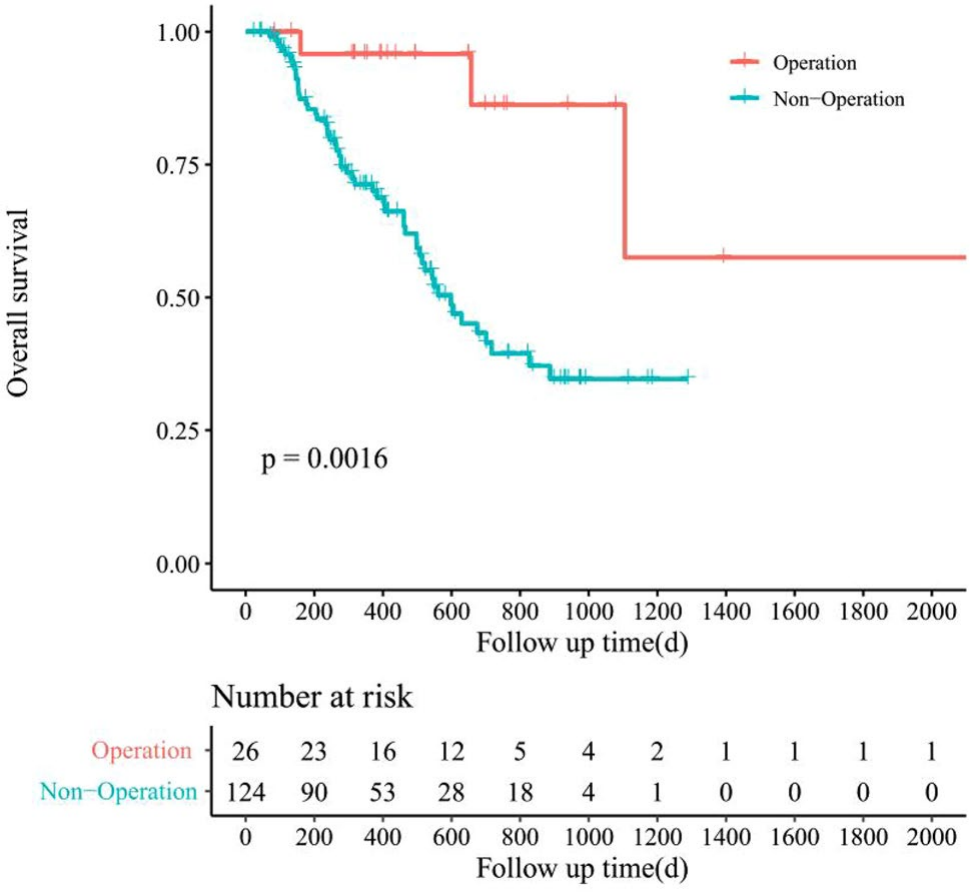
Kaplan–Meier survival curve for overall survival in CNLC IIIb stage HCC patients with surgical treatment or not after achieved PR/CR after conversion therapy.

**Figure 4:**
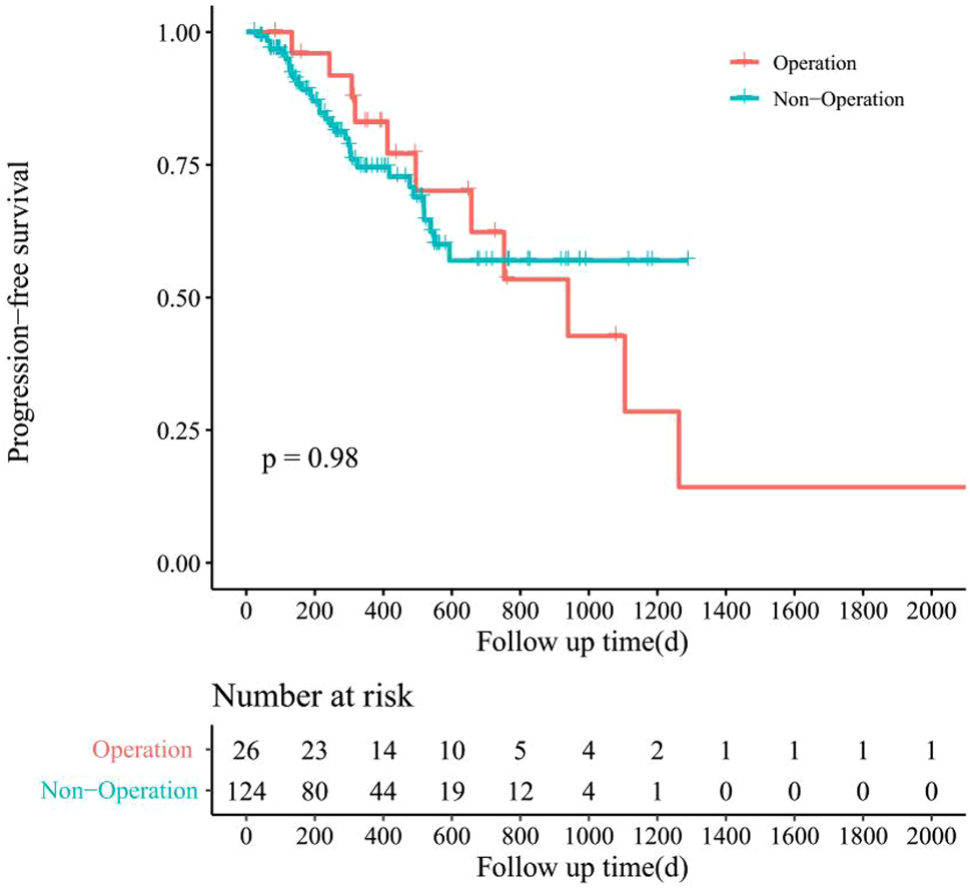
Kaplan–Meier survival curve for progress-free survival in CNLC IIIb stage HCC patients with surgical treatment or not after achieved PR/CR after conversion therapy.

The survival curve for OS indicated a longer survival duration among patients who underwent surgical treatment (see Fig. 3). However, there was no significant disparity in PFS between the two groups (see Fig. 4). Although the median OS was significantly extended in the surgical group, there was no notable variance in median PFS between the groups.

The discrepancy between OS and PFS implies that while surgical intervention may prolong patient survival, it does not necessarily correlate with an extended period before disease progression. Specifically, within the surgical group, 11 patients experienced disease progression, primarily manifested as intrahepatic recurrence.

### Recurrence pattern for recurrence patients

Out of 22 patients who experienced postoperative recurrence, 15 had intrahepatic recurrence, and 7 had extrahepatic recurrence. The remaining recurrence cases are detailed in Table 5.

**Table 5:** Recurrence patterns.

	Patients
Recurrence	22
Recurrence type	
Intrahepatic	15
Extrahepatic	7
PVTT	2
BCLC stage	
0-B	14
C-D	8

## Discussion

China has a notably high incidence of liver cancer, particularly HCC, with a mortality rate nearly double that of global statistics^10^. The unsatisfactory treatment outcome for HCC patients is mainly lied on the high number of patients with advanced stage and limited treatment options available for these patients^11^. In recent years, significant progress has been made in drug therapy, interventional therapy and radiotherapy for HCC. An increasing number of successful cases of translational therapy have been reported and combination therapy has been widely adopted. Firstly, the effectiveness of first-line drug therapy has been further confirmed. Sorafenib or lenvatinib in combination with atelizumab or bevacizumab have become the primary treatment regimens in common use recently^12–14^. Additionally, ZhangY et al. noted in their study that 82 of 831 patients with unresectable HCC treated with TACE met the criteria for resectability^15^. Sato et al. reported a case of tumor down-staging and successful surgical resection after lenvatinib combined with TACE therapy^16^; Takeda et al. reported successful conversion of unresectable hepatocellular carcinoma to resectable using regrafinib as conversion therapy^17^. Furthermore, it has been demonstrated in several studies that radiation-based combination therapy regimens may also lead to successful conversion of patients with unresectable HCC^18–21^. The above treatment regimen has also been applied to the translational treatment of CNLC stage IIIb patients with good results in some patients. However, once this group of patients has achieved PR/CR and become stabilized, a question arises as to whether they should continue maintenance therapy or undergo surgical treatment to resect the tumor? There is a lack of evidence on this issue worldwide. Only Xiaobo Yang et al. reported 9 cases of successful surgical resection of CNLC stage IIIb HCC patients after conversion therapy^22^. The purpose of this study is to provide evidence-based medical experience on whether surgical treatment can benefit this group of patients and to provide guidance for the treatment CNLC stage IIIb HCC.

For HCC patients with extrahepatic metastases, although systemic treatments aimed at tumor down-staging can significantly increase the number of surgically treatable patients^23^, surgical treatment is not recommended by clinical guidelines at this stage. Therefore, the number of patients receiving surgical treatment is extremely low. Thus, a multicenter, real-world study was conducted to ensure the integrity and authenticity of this study. In this study, all enrolled patients underwent conversion therapy and their primary tumors were assessed as PR/CR and none of the metastatic lesions showed progressive manifestations, although they received diverse conversion therapies. The baseline data showed no statistical differences between the two groups at the starting point, which demonstrated that the general and tumor conditions of the two groups were approximately the same and comparable. Patients who underwent surgery after conversion therapy achieved a longer OS compared to those who did not. However a slight regret is that no significant difference in PFS was revealed. We thought there are several possible reasons: First of all, overall survival is recognized as the "gold standard" for efficacy evaluation and provides a more objective prognosis of patients, while PFS has its relative limitations. Secondly, adjuvant therapy for patients with advanced hepatocellular carcinoma is affirmed by plenty of researches, which demonstrated that patients receiving medical therapy, interventional therapy or radiotherapy postoperatively can achieve longer survival^24–26^. Patients included in this study received diverse adjuvant therapy after surgery, which to some extent may influence PFS and OS. Besides, for patients with CNLC stage IIIb HCC, although surgical resection can effectively reduce the patient's tumor burden, most patients are still in a state of high tumor burden, which may lead to a high rate of recurrence for these patients^27^. Furthermore, surgery is not a very effective solution for metastases, except for lymph node metastases, which are mostly removed during surgery. Lung and bone metastases are often treated postoperatively with radio frequency ablation or radiotherapy and adjuvant therapy is generally not administered during the perioperative period. These metastases may promote tumor progression and impact PFS to some extent, although further research is needed. Finally, PFS can be affected by different follow-up intervals, so OS may be a more valuable index to assess the prognosis.

It is important to acknowledge the limitations of the present study. First, this is a retrospective study, bias may exist in patient selection. Also the resection group had a small number of patients. Despite our best efforts to collect such kind of patients across the country, the number of patients was still limited as surgery was not a standard treatment for advanced HCC. Future studies will aim to expand the cohort and provide high-level evidence. Additionally, the follow-up period was not long enough for surgical patients, which may have impacted the outcome. We will continue to follow up with more accurate results over a longer period of time. Furthermore, the different treatment options for metastases among the patients in this study may have also impacted the prognosis. Notably, lung metastases, whose P value is quite close to the boundary of significance in the univariate analyses, may led to a shorter PFS for CNLC stage IIIb HCC patients. A prospective study is needed to evaluate the effect of surgical resection in patients with extrahepatic metastasis and treated with systemic therapies or multi-modality treatment.

## Conclusions

In summary, HCC patients with extrahepatic metastases may obtain longer overall survival by receiving surgical treatment after achieving PR/CR with conversion therapy. Among these patients, portal vein invasion is an independent risk factor that leads to a poor prognosis. Effective first-line treatments and surgical treatments are protective factors for these patients. Nevertheless, further research is necessary in the future and we are confident that these findings will pave the way for more effective treatments for HCC patients with extrahepatic metastases.

## Data Availability

The datasets generated from this research can be disclosed only in specific circumstances. Further inquiries can be directed to the corresponding author.
